# *In Vitro* and *In Vivo* Antifungal Profile of a Novel and Long-Acting Inhaled Azole, PC945, on Aspergillus fumigatus Infection

**DOI:** 10.1128/AAC.02280-16

**Published:** 2017-04-24

**Authors:** Thomas Colley, Alexandre Alanio, Steven L. Kelly, Gurpreet Sehra, Yasuo Kizawa, Andrew G. S. Warrilow, Josie E. Parker, Diane E. Kelly, Genki Kimura, Lauren Anderson-Dring, Takahiro Nakaoki, Mihiro Sunose, Stuart Onions, Damien Crepin, Franz Lagasse, Matthew Crittall, Jonathan Shannon, Michael Cooke, Stéphane Bretagne, John King-Underwood, John Murray, Kazuhiro Ito, Pete Strong, Garth Rapeport

**Affiliations:** a Pulmocide Ltd., London, United Kingdom; b Institut Pasteur, CNRS, Molecular Mycology Unit, French National Reference Center for Invasive Mycoses & Antifungals, URA3012, Paris, France; c Paris Diderot, Sorbonne Paris Cité University, Paris, France; d Parasitology-Mycology Laboratory, Saint Louis Hospital, Assistance Publique-Hôpitaux de Paris (APHP), Paris, France; e Centre for Cytochrome P450 Biodiversity, Institute of Life Science, Swansea University Medical School, Wales, United Kingdom; f Nihon University, Chiba, Japan; g Sygnature Discovery Ltd., Nottingham, United Kingdom; h Compchem Resource, Pendock, United Kingdom

**Keywords:** Aspergillus fumigatus, azole, inhalation, CYP51, azole resistant, long acting

## Abstract

The profile of PC945, a novel triazole antifungal designed for administration via inhalation, was assessed in a range of *in vitro* and *in vivo* studies. PC945 was characterized as a potent, tightly binding inhibitor of Aspergillus fumigatus sterol 14α-demethylase (CYP51A and CYP51B) activity (50% inhibitory concentrations [IC_50_s], 0.23 μM and 0.22 μM, respectively) with characteristic type II azole binding spectra. Against 96 clinically isolated A. fumigatus strains, the MIC values of PC945 ranged from 0.032 to >8 μg/ml, while those of voriconazole ranged from 0.064 to 4 μg/ml. Spectrophotometric analysis of the effects of PC945 against itraconazole-susceptible and -resistant A. fumigatus growth yielded IC_50_ (determined based on optical density [OD]) values of 0.0012 to 0.034 μg/ml, whereas voriconazole (0.019 to >1 μg/ml) was less effective than PC945. PC945 was effective against a broad spectrum of pathogenic fungi (with MICs ranging from 0.0078 to 2 μg/ml), including Aspergillus terreus, Trichophyton rubrum, Candida albicans, Candida glabrata, Candida krusei, Cryptococcus gattii, Cryptococcus neoformans, and Rhizopus oryzae (1 or 2 isolates each). In addition, when A. fumigatus hyphae or human bronchial cells were treated with PC945 and then washed, PC945 was found to be absorbed quickly into both target and nontarget cells and to produce persistent antifungal effects. Among temporarily neutropenic immunocompromised mice infected with A. fumigatus intranasally, 50% of the animals survived until day 7 when treated intranasally with PC945 at 0.56 μg/mouse, while posaconazole showed similar effects (44%) at 14 μg/mouse. This profile affirms that topical treatment with PC945 should provide potent antifungal activity in the lung.

## INTRODUCTION

The current management of the three major forms of aspergillosis, invasive aspergillosis (IA), chronic pulmonary aspergillosis (CPA), and allergic bronchopulmonary aspergillosis (ABPA) (1–4), involves prophylactic or therapeutic administration of triazoles and, infrequently, surgical intervention (5). Existing antifungal medicines are predominantly dosed either orally or systemically. These frequently exploited routes of delivery are poor for treating airway disease, since drug concentrations achieved at the site of infection tend to be lower than those in other, healthy organs. This is especially so for the liver, which is a site of triazole toxicity: up to 15% of patients treated with voriconazole experience raised transaminase levels (6, 7). Exposure of the liver also results in significant drug interactions arising from triazole inhibition of hepatic P450 enzymes (8, 9).

**FIG 1:**
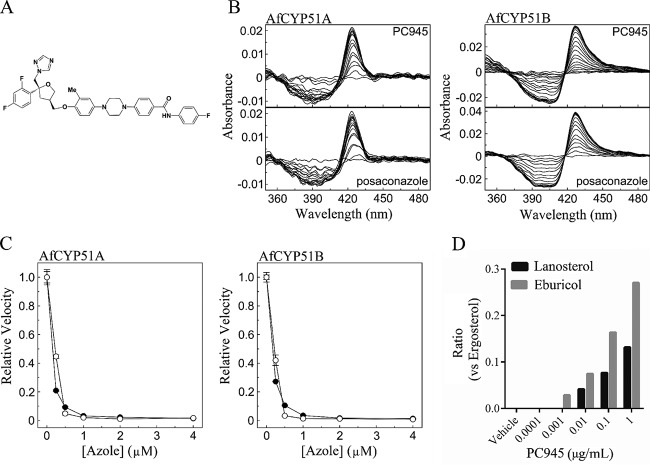
Efficacy of PC945 as an inhibitor of A. fumigatus sterol 14α-demethylases (CYP51 enzymes). (A) Structure of PC945. (B) Type II azole binding spectra for A. fumigatus CYP51A and CYP51B. Each experiment was performed 4 to 6 times, although data for only one replicate are shown. (C) Azole IC_50_ determinations for posaconazole (●) and PC945 (○). Mean relative velocity values are shown with standard deviations. (D) Sterol composition of A. fumigatus treated with PC945. The relative levels of lanosterol and eburicol are shown.

It is evident that there is an unmet clinical need for improved antifungal therapies which elicit fewer drug interactions, show reduced toxicity, achieve higher and more sustained pulmonary drug concentrations, and also demonstrate potent activity against azole-resistant Aspergillus strains. Thus, there are several advantages of topical treatment over oral/systemic treatment which alter the risk-benefit ratio of treatment favorably. An optimized compound for topical delivery should have prolonged lung tissue residence with limited systemic exposure to display a better adverse effect profile and to eradicate invasive aspergillosis due to a high-concentration exposure. We have undertaken an extensive lead optimization program in order to identify potent azole antifungal agents with optimal properties for topical administration to the lung, including tissue retention and physicochemical properties required for formulation. In this report, we disclose the *in vitro* and *in vivo* activity of PC945, which has the chemical formula 4-[4-(4-{[(3*R*,5*R*)-5-(2,4-difluorophenyl)-5-(1*H*-1,2,4-triazol-1-ylmethyl)oxolan-3-yl]methoxy}-3-methylphenyl)piperazin-1-yl]-*N*-(4-fluorophenyl)benzamide (Fig. 1A) and is a novel triazole antifungal agent designed specifically for inhaled administration (10).

## RESULTS

### CYP51-binding properties.

PC945 produced type II difference spectra when titrated against purified Aspergillus fumigatus CYP51A and CYP51B enzymes (AfCYP51A and AfCYP51B) and bound to CYP51A with an affinity similar to that of posaconazole (Table 1; Fig. 1B). In contrast, in ligand titration experiments with purified CYP51B, PC945 yielded a sigmoid binding saturation curve, while posaconazole gave the expected tight-binding saturation curve (see Fig. S1 in the supplemental material). A modified two-site allosteric model gave the best “off-the-shelf” fit of the sigmoid PC945 saturation curve, yielding dissociation constant (*K_d_*1 and *K_d_*2) values of 19,298 μM and 0.32 μM, respectively. This positive cooperative allosterism suggests either the existence of two nonequivalent ligand binding sites or the existence of two different binding conformations/orientations for the PC945 molecule within CYP51B that are responsible for the generation of the type II difference spectrum. Posaconazole bound tightly to purified CYP51B, with a *K_d_* value of 0.012 μM.

**TABLE 1:** Azole *K_d_* and IC_50_ determinations versus A. fumigatus CYP51 (AfCYP51) enzymes

Test agent	Parameter value (μM)^*a*^				
	AfCYP51A		AfCYP51B		
	*K_d_*	IC_50_	*K_d_*1	*K_d_*2	IC_50_
PC945	0.50	0.23	19,298	0.32	0.22
Posaconazole	0.96	0.16	0.012	NA	0.17

a *K_d_* determinations used 4 μM purified AfCYP51A and 4 μM AfCYP51B. IC_50_ determinations used 0.5 μM AfCYP51A and 0.5 μM AfCYP51B, recovered from the membrane fraction of E. coli expression clones. NA, not applicable.

### Inhibitory activity against A. fumigatus CYP51 enzymes.

The inhibitory activities of PC945 and posaconazole against A. fumigatus sterol 14α-demethylases were determined using 0.5 μM AfCYP51A and 0.5 μM AfCYP51B from the membrane fraction prepared from Escherichia coli expression clones. Both PC945 and posaconazole were strong, tightly binding inhibitors of CYP51A and CYP51B *in vitro* activity (Table 1; Fig. 1C), suggesting *K_i_*_.app_ values of <1 nM for both compounds (11). Moreover, PC945 was as effective as posaconazole, and both agents appeared to share the same mode of action, i.e., directly coordinating as the sixth axial ligand of the CYP51 heme iron. No allosterism was observed during the inhibition of AfCYP51B activity by PC945.

### Cell-based A. fumigatus sterol composition and CYP51 assay.

Analysis of sterol composition was performed by gas chromatography-mass spectrometry (GC-MS). Treatment with increasing concentrations of either posaconazole or PC945, from 0 to 1 μg ml^−1^, resulted in the dose-dependent accumulation of the 14α-methylated sterols (lanosterol and eburicol) and the corresponding depletion of the final sterol product, i.e., ergosterol (Table 2; Fig. 1D).

**TABLE 2:** Sterol composition of A. fumigatus treated with either posaconazole or PC945

Sterol	Sterol content in cells (%)											
	DMSO-treated cells	Posaconazole-treated (μg/ml) cells					DMSO-treated cells	PC945-treated (μg/ml) cells				
		0.0001	0.001	0.01	0.1	1		0.0001	0.001	0.01	0.1	1
Ergosterol	100	94.5	87.2	74.7	67.8	67.4	100	95.9	94.7	86.7	80.6	71.3
Ergost-5,7-dienol	0	3.3	3.9	0	0	0	0	4.1	2.5	3.3	0	0
Lanosterol	0	0	3.0	7.0	8.8	8.8	0	0	0	3.6	6.2	9.4
Eburicol	0	2.2	5.9	18.3	23.4	23.8	0	0	2.7	6.5	13.2	19.3

We also investigated enzyme-inhibitory activity in a plate-based A. fumigatus cell-based ergosterol assay. This test system takes advantage of the fact that cholesterol oxidase can utilize ergosterol as a substrate, with a 65% loss of sensitivity. Oxidation of ergosterol was determined by observing the conversion of the weakly fluorescent compound resazurin to the highly red fluorescent compound resorufin and was normalized using crystal violet staining. With an inhibitor activity resembling that in the cell-free model of CYP51, PC945 strongly inhibited ergosterol production (50% inhibitory concentration [IC_50_] = 0.0047 μg/ml [0.0069 μM]) and was 14- and 2.6-fold more potent than voriconazole (IC_50_ = 0.067 μg/ml [0.19 μM]) and posaconazole (IC_50_ = 0.012 μg/ml [0.017 μM]), respectively.

### *In vitro* antifungal activity against azole-susceptible and azole-resistant strains of A. fumigatus.

The concentrations of test agents required to achieve 50% inhibition (IC_50_) and 90% inhibition (IC_90_) (both determined based on optical density [OD]) of the growth of a number of A. fumigatus strains (itraconazole-susceptible strains NCPF2010, AF294, and AF293 and itraconazole-resistant strains AF72, AF91, and TR34/L98H) were calculated from growth curves generated using a modified 384-well EUCAST microdilution method and compared to positive and negative controls. Overall, PC945 was more active than all reference compounds, including voriconazole, posaconazole, and itraconazole, against itraconazole-susceptible A. fumigatus strains (NCPF2010, AF294, and AF293) (12, 13) (Table 3). In addition, PC945 was the most active test agent against known itraconazole-resistant A. fumigatus strains (AF72 and AF91) (14, 15) (Table 3). Against the A. fumigatus strain TR34/L98H, containing the environmentally acquired TR34/L98H mutation (16), PC945, voriconazole, itraconazole, and caspofungin all failed to achieve 90% inhibition of fungal growth, while posaconazole displayed an IC_90_ value of 0.13 μg/ml. However, PC945 achieved an IC_50_ of 0.034 μg/ml against this strain, thereby revealing it to be 2.5-fold more potent than posaconazole (Table 3). Bovine serum albumin (BSA) supplementation was confirmed to have no or little effect on the MICs of voriconazole (2 μg/ml without BSA, 1 to 2 μg/ml with BSA), posaconazole (0.03 μg/ml without BSA, 0.06 μg/ml with BSA), itraconazole (0.25 μg/ml without BSA, 0.125 μg/ml with BSA), and amphotericin B (1 μg/ml without BSA, 2 μg/ml with BSA), using A. fumigatus quality control strain ATCC 204305, but it showed marginal effects on the MIC of PC945, a more lipophilic compound (0.25 μg/ml without BSA, 0.0625 μg/ml with BSA).

**TABLE 3:** Antifungal effects of PC945 and known antifungal agents in azole-susceptible and azole-resistant strains of A. fumigatus^*a*^

Strain	Resistance mechanism	IC_50_ (IC_90_) (μg/ml) of indicated agent					
		PC945	Voriconazole	Posaconazole	Itraconazole	Amphotericin B	Caspofungin
NCPF2010	None	0.0084 (0.010)	0.16 (0.20)	0.0086 (0.014)	0.057 (0.085)	0.23 (0.48)	0.11 (>1)
AF294	None	0.0020 (0.0043)	0.082 (0.27)	0.0056 (0.011)	0.041 (0.052)	0.21 (0.79)	>1 (>1)
AF293	None	0.0012 (0.0041)	0.25 (0.74)	0.010 (0.028)	0.032 (0.23)	0.24 (0.85)	>1 (>1)
AF72	G54E mutation	0.0061 (0.029)	0.019 (0.062)	0.032 (0.19)	0.43 (>1)	0.18 (0.64)	0.10 (>1)
AF91	M220V mutation	0.0081 (0.059)	0.12 (0.38)	0.024 (0.12)	0.26 (>1)	0.42 (>1)	0.072 (>1)
TR34/L98H	TR34/L98H mutation	0.034 (>1)	>1 (>1)	0.086 (0.13)	0.22 (>1)	0.14 (0.29)	0.082 (>1)

a IC_50_ and IC_90_ values were determined from optical density measurements.

### *In vitro* antifungal activity against clinically isolated A. fumigatus strains.

Test agents were evaluated on 50 clinical isolates obtained from the St. Louis Hospital, Paris, France, and on 46 clinical isolates obtained from the NW Mycology Centre, United Kingdom. Based on the geometric mean, PC945 was found to be 2.5-fold more potent than voriconazole but comparable to posaconazole (Table 4). Among the clinical isolates from the NW Mycology Centre, 13 of the 46 strains were found to be posaconazole resistant, 7 of the 13 posaconazole-resistant isolates were confirmed to be itraconazole resistant, and 2 of those were pan-azole resistant, including resistance to voriconazole, based on the EUCAST epidemiological cutoff value (ECOFF). For 5 of the posaconazole-resistant isolates, PC945 did not inhibit growth completely at concentrations of up to 8 μg/ml. During this assay, the quality control strain A. fumigatus ATCC 204305 was used for validation. For this strain, posaconazole showed a MIC of 0.25 μg/ml, within the range set by the EUCAST guidelines.

**TABLE 4:** *In vitro* activities of PC945, posaconazole, and voriconazole against 96 clinically isolated A. fumigatus strains^*a*^

Test agent	MIC (μg/ml)^*b*^				
	Range	Geometric mean	Mode	MIC_50_	MIC_90_
PC945	0.032–>8	0.17*	0.125	0.125	1
Voriconazole	0.064–4	0.42	0.5	0.5	1
Posaconazole	0.016–2	0.1	0.032	0.063	0.5

a All MICs were determined visually.

b *, *P* < 0.05 for PC945 versus the results for posaconazole (one-way ANOVA with Tukey's test).

### *In vitro* assessment of antifungal activity by use of CLSI methodology.

Visual assessment of the growth of four itraconazole-susceptible A. fumigatus strains demonstrated that PC945 was the most potent compound tested, with an MIC value of 0.031 μg/ml, while voriconazole and posaconazole were less effective (Table 5). Thus, the superiority of PC945 over voriconazole for A. fumigatus growth inhibition was confirmed by the CLSI method as well as the EUCAST microdilution method.

**TABLE 5:** Antifungal effects of PC945 and known antifungal agents on four itraconazole-susceptible A. fumigatus strains, as determined using CLSI methodology

Test agent	MIC (μg/ml)^*a*^^,^^*c*^		MIC_50_ (μg/ml)^*b*^^,^^*c*^	
	Median	Interquartile range	Median	Interquartile range
PC945	0.031*	0.020–0.031	0.011**	0.0083–0.024
Voriconazole	0.5	0.5–0.5	0.14	0.082–0.15
Posaconazole	0.047	0.031–6.0	0.015	0.0095–0.016

a Determined visually.

b Determined using optical density measurements.

c *, *P* < 0.05; **, *P* < 0.01 for PC945 versus the results for voriconazole (Kruskal-Wallis one-way ANOVA with Dunn's test).

### Antifungal activity against non-A. fumigatus species.

The *in vitro* activities of PC945, voriconazole, and posaconazole against 22 pathogenic fungi (1 or 2 isolates each) are displayed in Table 6. The data for Aspergillus terreus show it to be more susceptible to PC945 than to posaconazole. In addition, PC945 was found to have antifungal activity against Aspergillus carbonarius and Aspergillus flavus, although it was less potent than posaconazole or voriconazole. Against Candida albicans (both azole-susceptible and azole-resistant strains), Candida glabrata, and Candida krusei, PC945 was generally more active than voriconazole and shared equal potency with posaconazole. PC945, voriconazole, and posaconazole were comparable in their effectiveness against Trichophyton rubrum. The remarkable potency of PC945 against Rhizopus oryzae was seen in a greatly improved MIC (2 μg/ml) compared to that of voriconazole and posaconazole (>8 μg/ml). The potencies of PC945 against Cryptococcus neoformans and Cryptococcus gattii were higher than or similar to those of voriconazole and posaconazole. In contrast, Aspergillus niger, Aureobasidium pullulans, Penicillium chrysogenum, Penicillium citrinum, Cladosporium argillaceum, Chaetomium globosum, Gibberella zeae (Fusarium graminearum), Lichtheimia corymbifera, Mucor circinelloides, and Rhizomucor pusillus were not susceptible to PC945 treatment within the concentration range tested.

**TABLE 6:** Antifungal effects of PC945 and posaconazole on other fungal species

Species (strain[s])	No. of strains tested	Culture method	MIC (μg/ml)^*a*^		
			PC945	Voriconazole	Posaconazole
Aspergillus carbonarius (ATCC 8740)	1	CLSI	4	0.5	0.063
Aspergillus flavus (ATCC 204304)	1	CLSI	>8	2	0.13
Aspergillus flavus (AFL8, NRRC3357)	2	EUCAST	6	0.63	0.16
Aspergillus niger (ATCC 1015)	1	EUCAST	>8	1	0.20
Aspergillus terreus (AT49, AT7130)	2	EUCAST	0.078	1	0.093
Penicillium chrysogenum (ATCC 9480)	1	CLSI	>8	2	0.13
Penicillium citrinum (ATCC 9849)	1	CLSI	>8	>8	0.5
Trichophyton rubrum (ATCC 10218)	1	CLSI	0.031	0.063	0.031
Aureobasidium pullulans (ATCC 9348)	1	CLSI	>8	>8	1
Cladosporium argillaceum (ATCC 38013)	1	CLSI	>8	0.5	0.25
Candida albicans^*b*^ (20240.047, ATCC 10231)	2	CLSI	0.081	0.14	0.081
AR Candida albicans^*b*^^,^^*c*^ (20183.073, 20186.025)	2	CLSI	8.25	10	8.13
Candida glabrata^*b*^ (ATCC 36583, R363)	2	CLSI	0.5	8.13	0.5
Candida krusei (ATCC 6258)	1	CLSI	0.125	0.25	0.125
Chaetomium globosum (ATCC 44699)	1	CLSI	>8	1	0.25
Gibberella zeae (Fusarium graminearum) (ATCC 16106)	1	CLSI	>8	>8	>8
Cryptococcus gattii (clinical isolate)	1	EUCAST	0.25	0.125	0.5
Cryptococcus neoformans (ATCC 24067)	1	CLSI	0.008	0.016	0.016
Lichtheimia corymbifera (ATCC 7909)	1	CLSI	>8	>8	>8
Mucor circinelloides (ATCC 8542)	1	CLSI	>8	>8	>8
Rhizomucor pusillus (ATCC 16458)	1	CLSI	>8	>8	>8
Rhizopus oryzae (ATCC 11145)	1	CLSI	2	>8	>8

a Due to the limited number of strains tested, the mean isolate MICs are presented.

b The MIC given is the MIC_50_.

c AR, azole resistant (fluconazole and voriconazole).

### *In vitro* determination of persistence of action.

**FIG 2:**
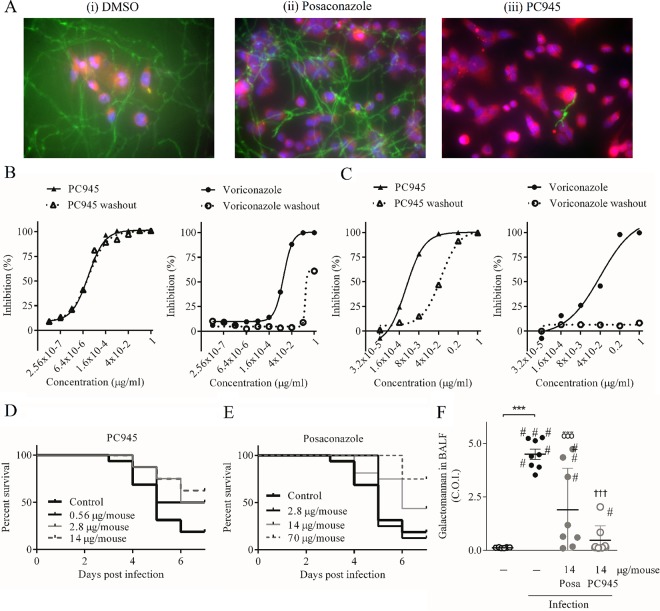
Antifungal activity of PC945 against A. fumigatus *in vitro* and *in vivo*. (A) A549 cells treated with PC945 or posaconazole and infected with GFP-expressing A. fumigatus. Green, GFP-expressing A. fumigatus; blue, DAPI-stained nucleus; red, CellTracker-stained cytoplasm. (B) Persistence of action of PC945 and voriconazole on A. fumigatus hyphae. (C) Persistence of action of PC945 and voriconazole on human bronchial cells (BEAS2B cells) infected with A. fumigatus. (D and E) Effects of once-daily intranasal treatment with PC945 (0.56, 2.8, or 14 μg/mouse) and posaconazole (2.8, 14, or 70 μg/mouse) on survival of A. fumigatus-infected immunocompromised mice (*n* = 8 to 16). (F) Galactomannan levels in BALF. Each horizontal bar shows the mean ± SEM for 8 mice per group. #, dead before day 7; ***, significant difference from no infection (*P* < 0.001); †††, significant difference from infection control (*P* < 0.001).

Retention of test agents within the hyphae of A. fumigatus was determined using a resazurin-based microtiter assay. The inhibition of fungal growth arising from continuous contact of the test compounds with A. fumigatus hyphae for 16 h was measured and compared to that obtained after contact with drug for only 20 min, followed by washout and incubation for the same period. As shown in Table 7 and Fig. 2B, the antifungal potencies of voriconazole and posaconazole diminished markedly after short contact and washout, by factors of >93-fold and 4.9-fold, respectively. In contrast, PC945 showed no change in its antifungal activity between wash and nonwash conditions in this experimental paradigm (Table 7). It was also observed that PC945 (IC_50_, 0.00010 μg/ml) was 110- and 4.5-fold more potent than voriconazole and posaconazole, respectively, at inhibiting hyphal A. fumigatus growth.

**TABLE 7:** Potencies and persistence of action of PC945, posaconazole, and voriconazole

Test agent	Hyphae			BEAS2B cells		
	IC_50_ (μg/ml)^*a*^		Fold change	IC_50_ (μg/ml)		Fold change
	No washout	Washout		No washout	Washout	
PC945	0.00010*	0.000086	0.87	0.0037	0.043	11.5
Voriconazole	0.011	>1	>93	0.054	>1	>18.6
Posaconazole	0.00045	0.0022	4.90	0.0031	0.046	14.7

a *, *P* < 0.05 for PC945 versus the results for voriconazole (Kruskal-Wallis one-way ANOVA with Dunn's test).

In the second system, the persistence of action of PC945 was quantified using galactomannan (GM) production in the supernatant as an index of fungal growth. BEAS2B cells were infected with A. fumigatus, and the effects of a 24-h washout period prior to infection were examined. A 1-h contact time with PC945 followed by a 24-h washout resulted in an approximately 11-fold loss of potency against A. fumigatus compared to that in the control assay in which there was no washout. Although posaconazole showed a similar or slightly greater loss of potency on washout, it was notable that voriconazole was ineffective after a 24-h washout. These data imply that only a short period of contact of bronchial epithelial cells with PC945 would be required for the agent to exhibit a long duration of therapeutic action (Table 7; Fig. 2C).

### *In vivo* antifungal activity.

The potential of intranasally administered PC945 as a daily therapeutic treatment for pulmonary Aspergillus infection was investigated using immunocompromised, temporarily neutropenic mice. In this model, 81% of control mice (13/16 mice) were dead or had been removed from the study (“dropped out”) by day 6 postinfection, and only 19% of mice survived (Table 8; Fig. 2D). However, we observed that 50, 63, and 63% of mice dosed with PC945 (0.56, 2.8, and 14 μg/mouse [intranasal application of 0.016-, 0.08-, and 0.4-mg/ml suspensions, respectively]) survived (Table 8; Fig. 2D). In contrast, although the highest dose (70 μg/mouse) gave a 75% survival rate, the effects of posaconazole at 2.8 and 14 μg/mouse were weaker than that of PC945 at 0.56 μg/mouse (Table 8; Fig. 2E). Furthermore, the GM level in bronchoalveolar lavage fluid (BALF) collected on day 7 postinfection or on the day that an animal dropped out of the study was significantly reduced by treatment with PC945, to a superior degree over that obtained with posaconazole treatment (Fig. 2F). Intranasal A. fumigatus infection is known to cause “rolling” behavior in mice due to central nervous system (CNS) effects via respiratory-systemic infection (17). In this study, it was noted that PC945, but not posaconazole, substantially inhibited the incidence of this rolling behavior (Fig. S2).

**TABLE 8:** *In vivo* activities of PC945 and posaconazole

Test agent	Dose (μg/mouse)	No. of survivors on day 7/total no. of mice (%)	Median days of survival	Log rank (Mantel-Cox test) *P* value^*a*^
Vehicle		3/16 (19)	5	
PC945	0.56	4/8 (50)	6	0.14
	2.8	5/8 (63)	6.5	0.022*
	14	10/16 (63)	Undefined	0.0095**
Posaconazole	2.8	1/8 (13)	5	0.69
	14	7/16 (44)	6	0.050*
	70	6/8 (75)	Undefined	0.0028**

a *, *P* < 0.05; **, *P* < 0.01.

## DISCUSSION

It has been demonstrated herein that PC945 is a potent antifungal agent that possesses activity against a broad range of both azole-susceptible and azole-resistant strains of A. fumigatus. Against itraconazole-susceptible A. fumigatus strains, PC945 showed an increase in potency between 20- and 180-fold over that of voriconazole, with a comparable or improved potency versus that of posaconazole. In an itraconazole-resistant strain of A. fumigatus with a CYP51A M220V mutation, obtained from a patient undergoing high-dose itraconazole therapy, and an A. fumigatus strain harboring the G54E mutation, recently discovered to be acquired environmentally (12, 18), PC945 was 6- and 2-fold more potent than voriconazole and posaconazole, respectively. Furthermore, in a strain of A. fumigatus isolated in France (16) and displaying the environmentally acquired TR34/L98H mutation, PC945 was more active than voriconazole, although it did not achieve 90% inhibition of growth.

Against 96 clinically isolated A. fumigatus strains obtained from St. Louis Hospital, Paris, France, and the NW Mycology Centre, United Kingdom (Evotec UK Ltd.), the MIC range for PC945 was found to be 0.032 to >8 μg/ml, with a geometric mean of 0.17 μg/ml, and the MIC_50_ and MIC_90_ values were 0.125 and 1.0 μg/ml, respectively. The potency of PC945 was superior to that of voriconazole and comparable to that of posaconazole. It is noteworthy that out of 46 clinical isolates of A. fumigatus from the NW Mycology Centre, 13 strains were posaconazole resistant and 2 were pan-azole resistant, including having voriconazole resistance, based on the EUCAST ECOFF. Several reports have demonstrated that there is an increasing incidence of itraconazole-resistant A. fumigatus in the northwestern region due to extensive clinical use of itraconazole in this area of the United Kingdom (19, 20). PC945 showed inhibitory activity against 8 of the 13 azole-resistant strains but did not completely inhibit the growth of 5 strains when used at concentrations of up to 8 μg/ml. Although the strains are clearly resistant to azoles, the genetic cause(s) underlying resistance in these strains is unknown.

In this study, we modified the original EUCAST system. First, for our in-house screening regimen, we adapted the EUCAST methodology to a 384-well system, as this is a more efficient assay which has been shown for other fungal species to generate data comparable to those obtained in the 96-well assay (21). The same final concentration of dimethyl sulfoxide (DMSO) was applied across the plate to compare treatment effects with vehicle control effects more accurately; EUCAST currently suggests dilution of compounds with EUCAST medium, but some lipophilic or insoluble compounds may precipitate, generating misleading data. In fact, PC945 precipitated in EUCAST medium at >4 μg/ml, but voriconazole (less lipophilic) did not. Furthermore, data from Pulmocide and from the St. Louis Hospital in Paris were generated by supplementing the growth medium with 0.5% BSA, as this avoids the loss of lipophilic compounds bound to plastic surfaces during the assay and is not inhibitory to A. fumigatus growth (22, 23). Using the quality control strain A. fumigatus ATCC 204305, we confirmed that BSA supplementation did not affect the MICs of known antifungal agents, including voriconazole, posaconazole, itraconazole, and amphotericin B. Lastly, at Pulmocide we used turbidity, determined by assessment of OD by use of a spectrophotometer, as a measure of fungal growth to enable more accurate quantification of the inhibitory effects of treatments. All the modifications described above helped to quantify the antifungal activity of test agents more accurately.

While A. fumigatus represents a severe global health risk, other fungal species continue to be equally problematic. Invasive candidiasis affects 46,000 people each year in the United States alone, and an estimated 1 million people with HIV/AIDS contract cryptococcal meningitis worldwide annually (24, 25). These figures underline the pressing need for safer and more effective antifungals that deliver a broad spectrum of activity. This report discloses that the novel agent PC945 has a broad activity profile against a diverse range of fungal species. The growth of C. albicans, C. glabrata, and C. krusei was inhibited by PC945 as strongly as the inhibition with posaconazole and 1.7- to 16-fold more actively than the inhibition with voriconazole. Against C. neoformans, PC945 was 2-fold more potent than both voriconazole and posaconazole, while C. gattii was equally susceptible to the inhibitory activities of PC945, voriconazole, and posaconazole. Mucormycosis caused by R. oryzae has a mortality rate of 76% for patients with pulmonary infections (26). In this study, PC945 was particularly effective against R. oryzae (MIC value of 2.0 μg/ml), while voriconazole and posaconazole had no effect within the concentration range tested.

The proposed mechanism of action of PC945 is inhibition of sterol 14α-demethylase (CYP51A1), the enzyme required to convert eburicol to 14-demethylated eburicol, an essential step in the ergosterol biosynthesis pathway in fungi. Type II binding spectra, which display an *A*_max_ at 423 to 430 nm and a broad trough at 386 to 412 nm, arise through a specific interaction in which the triazole N-4 nitrogen (posaconazole) or the imidazole ring N-3 nitrogen coordinates as the sixth axial ligand with the heme iron to form a low-spin CYP51-azole complex (27, 28). PC945 produced type II difference spectra when it was titrated against purified A. fumigatus CYP51A and CYP51B enzymes, and it bound with an affinity for CYP51A similar to that of posaconazole but yielded a sigmoid binding saturation curve against CYP51B. The latter binding characteristic of PC945 was not reflected in the compound's inhibition of CYP51B activity, suggesting a difference in the enzyme's conformation in solution and that adopted when the enzyme is incorporated into cell membranes. Furthermore, the strong inhibition of CYP51A activity observed with both PC945 and posaconazole, indicative of tightly binding inhibitors (with an IC_50_ approximately half the enzyme concentration present), was tighter than predicted by the calculated *K_d_* values from ligand binding studies using recombinant CYP51A, suggesting that the conformation of purified CYP51A in solution differs from that in cell membranes. In the sterol composition assay, treatment with increasing concentrations of either PC945 or posaconazole, from 0 to 1 μg ml^−1^, resulted in an accumulation of the 14-methylated sterols, lanosterol and eburicol, and depletion of the final sterol product, ergosterol; this is consistent with CYP51 inhibition as the key pharmacological activity of both agents. In addition, the A. fumigatus cell-based ergosterol assay demonstrated that PC945 was 14- and 2.6-fold more potent at inhibiting ergosterol production than voriconazole and posaconazole, respectively. Thus, the mechanism of action of PC945, as for other triazole antifungals, is the inhibition of fungal sterol 14α-demethylase, resulting in the depletion of ergosterol in the fungal membrane and thereby disrupting membrane structure and function and inhibiting growth of the pathogenic organism (29).

A highly desirable feature of topical medicines is a long duration of action ensuring that the desired therapeutic activity is maintained throughout the interdose period. In order to explore this parameter, the persistence of action of PC945 was studied in a number of *in vitro* systems. For A. fumigatus hyphae, the IC_50_ measured for PC945 following a 20-min contact period and washout for 16 h was almost unchanged relative to that obtained following continuous contact with the drug for the same period without washout. Furthermore, in the BEAS2B cell line, washout for 24 h after a 1-h contact period resulted in only an approximately 10-fold loss of potency against A. fumigatus compared to that of the control, in which there was no washout period. This property of cellular persistence in the absence of the pathogen may be a particularly valuable property by enhancing the potential use of PC945 in prophylaxis.

The anti-Aspergillus activity of PC945 administered intranasally was also investigated in mice by use of a survival readout. PC945 was dosed to animals pretreated with a single round of cyclophosphamide and three rounds of hydrocortisone to induce temporary neutropenia, followed 24 h later by inoculation with A. fumigatus. In this study, 81% of vehicle-treated, A. fumigatus-infected mice were classified as dead/dropped out by day 7. However, once-daily treatment with low-dose PC945 showed marked beneficial effects on survival. While 44% of infected mice survived to day 7 when treated with posaconazole at 14 μg/mouse, those treated with PC945 showed 50% survival at a 25-fold lower dose (0.56 μg/mouse). Despite our data displaying similar antifungal activities as determined by the broth microdilution assay, these results indicate that PC945 significantly outperforms posaconazole *in vivo*. This superior profile probably arises from two factors, the first pharmacokinetic and the second pharmacodynamic. As discussed earlier, PC945 exhibits a longer duration of action than that of posaconazole, and the molecule is retained within the lung, such that little systemic exposure compared to that of posaconazole results after intranasal treatment (unpublished data). As a clinical strategy, topical treatment of the lung is advantageous over oral or intravenous therapy because it delivers high concentrations of an antifungal agent directly to the site of infection and avoids unfavorable systemic side effects. The benefits of inhaled administration for the treatment of invasive pulmonary aspergillosis have been shown in numerous studies involving amphotericin B, itraconazole, and voriconazole (30–32).

Development of resistance to antifungal therapy has been an increasing problem in recent years. It has been shown that a strategy for avoiding the development of resistance is to ensure that the ratio of treatment peak concentration to MIC is adequate (33). The relationship between resistance mutation induction and drug exposure has been well studied in bacteria. In the case of levofloxacin use against infection with Pseudomonas aeruginosa, exposure at an area under the concentration-time curve (AUC)/MIC ratio of 157 was calculated to prevent emergence of resistance (34). In the same manner, delivery of antifungals directly to the lung enables high AUC/MIC ratios to be achieved locally, reducing the risk of resistance.

PC945 is the first antifungal specifically designed as a once-daily, topical, inhaled treatment for Aspergillus infection of the lung. Designed to be retained within the target organ (such as the lung), treatment with PC945 results in very low systemic exposure (data not shown), thus reducing the potential risk for unwanted clinical effects. In addition, PC945 exhibits high levels of plasma protein binding, further reducing the likelihood of problems arising from circulating drug substance. Therefore, PC945 has the pharmacological and pharmaceutical properties to be a best-in-class, inhaled therapy for the treatment of A. fumigatus infection in humans.

## MATERIALS AND METHODS

### Antifungal agents.

For *in vitro* antifungal assays, stock solutions of test agents were prepared in DMSO (2,000 μg/ml). For *in vivo* studies, test agents were suspended in physiological saline. PC945 was synthesized by Sygnature Discovery Ltd. (Nottingham, United Kingdom), and voriconazole (Tokyo Chemical Industry UK Ltd., Oxford, United Kingdom), posaconazole (Apichem Chemical Technology Co., Ltd., Zhejiang, China), itraconazole (Arkopharma, Carros, France), amphotericin B (Selleckchem, Munich, Germany), and caspofungin (Selleckchem, Munich, Germany) were procured from commercial sources.

### A. fumigatus CYP51 binding assay and enzyme-inhibitory activity.

A. fumigatus CYP51 binding properties were determined as described by Warrilow et al. (35). Test agents were titrated against 4 μM recombinant A. fumigatus CYP51A or CYP51B protein, and binding saturation curves were constructed from the change in the absorbance between the spectral peak and the trough. A rearrangement of the Morrison equation was used to determine the dissociation constant (*K_d_*) values when ligand binding was tight (36).

A CYP51 reconstitution assay system was used to determine 50% inhibitory concentrations (IC_50_s) (37). Test agent was added to a mixture of 0.5 μM CYP51, 1 μM A. fumigatus cytochrome P450 reductase isoenzyme 1 (AfCPR1), 50 μM eburicol, 4% (wt/vol) 2-hydroxypropyl-β-cyclodextrin, 0.4 mg ml^−1^ isocitrate dehydrogenase, 25 mM trisodium isocitrate, 50 mM NaCl, 5 mM MgCl_2_, and 40 mM 3-(*N*-morpholino) propanesulfonic acid (MOPS) (pH ∼7.2). The mixtures were then incubated at 37°C for 10 min prior to initiation with 4 mM β-NADPHNa_4_ followed by shaking for 20 min at 37°C. Sterol metabolites were recovered by extraction with ethyl acetate followed by derivatization with 0.1 ml *N*,*O*-bis(trimethylsilyl)trifluoroacetamide:trimethylchlorosilane (99:1) and 0.3 ml anhydrous pyridine prior to analysis by gas chromatography-mass spectrometry.

### A. fumigatus sterol analysis.

A working suspension of A. fumigatus spores was prepared in filter-sterilized MOPS-RPMI 1640 (RPMI 1640 medium containing 2 mM l-glutamine, 2% glucose, and 0.165 M MOPS, buffered to pH 7 with NaOH) to a final concentration of 8 × 10^6^ spores ml^−1^. To each 100-mm petri dish, 10 ml of the working suspension was added, and the dishes were incubated for 4 h at 35°C and 5% CO_2_. Samples for baseline determinations were collected by scraping, pelleted by centrifugation at 2,000 rpm for 5 min, and stored at −80°C. Test compounds or DMSO (50 μl) was added to the remaining dishes, which were subsequently gently rocked by hand to disperse the compounds. Dishes were incubated for 2 h at 35°C and 5% CO_2_. Samples were collected and processed as described above. Posaconazole and PC945 concentrations of 0.0001, 0.001, 0.01, 0.1, and 1 μg ml^−1^ were tested. These samples were prepared in the laboratory at Pulmocide Ltd. and sent to the laboratory at the Centre for Cytochrome P450 Biodiversity, Institute of Life Science, Swansea University Medical School, for experimentation.

Nonsaponifiable lipids were extracted as previously reported (29) and were derivatized with 0.1 ml *N*,*O*-bis(trimethylsilyl)trifluoroacetamide:trimethylchlorosilane (99:1) and 0.3 ml anhydrous pyridine (2 h at 80°C) prior to analysis by gas chromatography-mass spectrometry (38). Sterol composition was calculated using peak areas from the gas chromatograms, and the mass fragmentation patterns compared to known standards were used to confirm sterol identity. The sterol contents of A. fumigatus (basal) and treated A. fumigatus (treated with either DMSO, posaconazole, or PC945) were determined.

### A. fumigatus cell-based ergosterol assay.

Growth medium (RPMI 1640, 2 mM l-glutamine, 2% glucose, 0.165 M MOPS, 0.5% BSA, pH 7.0) was added across a 96-well plate, and test agents were added in duplicate. A. fumigatus (NCPF2010) conidia were added across the plate to a final concentration of 1 × 10^4^ ml^−1^. After incubation for 24 h at 35°C, medium was removed from all wells and replaced with reaction buffer (Amplex red cholesterol assay kit; Thermo Fisher) and Amplex red solution. Plates were incubated for 30 min at 37°C, protected from light, after which fluorescence was quantified using a spectrophotometer. Medium was removed from all wells and replaced with crystal violet solution (1% [vol/vol]), and plates were incubated at room temperature on a shaker for 30 min. Plates were washed three times with phosphate-buffered saline (PBS), and sodium dodecyl sulfate solution (0.1% [vol/vol]) was added across the plate to lyse the cells. After incubation at room temperature for 1 h, the absorbance was measured by determining the OD_590_ by use of a spectrophotometer.

### Fluorescence imaging of A. fumigatus-infected cells.

Human alveolar epithelial cells (A549) were seeded onto collagen-coated coverslips and incubated at 37°C and 5% CO_2_ for 24 h. Cells were incubated in the presence of test agents for 2 h, after which the medium was replaced and the coverslips were incubated at 37°C and 5% CO_2_ for 24 h. CellTracker red CMTPX dye (Thermo Fisher) was added to the cell medium for 30 min, the wells were washed with PBS, and green fluorescent protein (GFP)-expressing A. fumigatus conidia (a kind gift from William Hope, University of Liverpool) were added to wells to a final concentration of 1 × 10^3^ spores ml^−1^. After 24 h of incubation at 35°C and 5% CO_2_, coverslips were washed and affixed to slides by use of Fluoroshield with DAPI (4′,6-diamidino-2-phenylindole) (Sigma).

### *In vitro* antifungal activity against A. fumigatus.

Assessment of antifungal activity against a selection of A. fumigatus laboratory/clinical strains (NCPF2010 [National Collection of Pathogenic Fungi {NCPF}, Bristol, United Kingdom], AF72 [NCPF, Bristol, United Kingdom], AF91 [NCPF, Bristol, United Kingdom], AF293 [NCPF, Bristol, United Kingdom], AF294 [NCPF, Bristol, United Kingdom], and TR34/L98H [Bretagne, St. Louis Hospital, Paris, France]) was performed according to the method in the European Committee on Antimicrobial Susceptibility Testing (EUCAST) definitive document EDef 9.3 (39), with the following exceptions: (i) 0.5% BSA was added to the growth medium to avoid any loss of lipophilic compounds by adherence to plastic plate surfaces and (ii) 384-well plates were used rather than 96-well plates. Growth medium (RPMI 1640, 2 mM l-glutamine, 2% glucose, 0.165 M MOPS, 0.5% BSA, pH 7.0) was added across the plate, test agents were added in quadruplicate, and the DMSO concentration was identical across the plates. Conidia were added across the plate to a final concentration of 1 × 10^5^ ml^−1^. Plates were incubated for 48 h at 35°C, after which turbidity was assessed by measuring the OD_530_ by use of a spectrophotometer, and the IC_50_ and IC_90_ values were calculated from the concentration-response curve generated for each test compound by use of a four-parameter logistic equation (Dotmatics, Bishops Stortford, United Kingdom). A. fumigatus ATCC 204305 was used as the assay control. Determination of antifungal activity against 50 A. fumigatus clinical isolates from St. Louis Hospital (Paris, France) was performed with 96-well plates, using the modified EUCAST method in the presence of 0.5% BSA as described above. Antifungal susceptibility testing of 46 A. fumigatus isolates (obtained from the NW England Mycology Reference Centre) was performed by Evotec UK Ltd. (Manchester, United Kingdom) according to EUCAST guidelines. Assessment of the antifungal activity against 4 of the A. fumigatus strains (ATCC 1028, ATCC 10894, ATCC 13073, and ATCC 16424) was performed by Eurofins Panlabs Taiwan Ltd. (Taipei, Taiwan) according to methodology described by the Clinical and Laboratory Standards Institute (CLSI).

### *In vitro* antifungal activity against other fungal species.

For the measurement of activity against Cryptococcus gattii, the method described in EUCAST definitive document EDef 7.2 was used, and assay plates were incubated statically at 37°C in ambient air for 24 h (±2 h) unless poor growth necessitated further incubation to 36 or 48 h (40). Antifungal potency against Aspergillus flavus, Aspergillus niger, and Aspergillus terreus was determined as set out in EUCAST definitive document EDef 9.2, and assay plates were incubated at 37°C for 48 h (41). These tests were conducted at Evotec UK Ltd. (Manchester, United Kingdom). Measurement of activity against other fungi was performed by Eurofins Scientific according to methodology described by the CLSI (document M38-A [42] or M27-A2 [43]).

### *In vitro* determination of persistence of action on A. fumigatus hyphae.

The persistence of action of test agents was calculated for A. fumigatus hyphae (NCPF2010). Conidia were diluted in growth medium (RPMI 1640, 2 mM l-glutamine, 2% glucose, 0.165 M MOPS, pH 7.0) and added across a 384-well plate to a final concentration of 1 × 10^3^/well. After incubation at 35°C for exactly 6 h, test and reference articles or undiluted DMSO (as vehicle) (0.5 μl/well) was added to the appropriate wells to give a final concentration of 0.5% DMSO. The plates were incubated for exactly 20 min at 35°C and 5% CO_2_. After the incubation time had elapsed, all wells on the designated washout plate were aspirated, and growth medium (100 μl/well) was added across the plate. For the nonwashout plate, no medium change was applied after compounds were added to hyphae. Resazurin (0.04%; diluted in growth medium) was added to all wells of both nonwashout and washout plates (5 μl/well) to give a final concentration of 0.002% resazurin. The plates were incubated at 35°C and 5% CO_2_ for 16 h. Subsequently, the fluorescence in each well was measured at 545/600 nm (λex/λem) by use of a multiscanner (Clariostar; BMG, Buckinghamshire, United Kingdom). The percent inhibition for each well was calculated, and the IC_50_ was calculated from the concentration-response curve generated for each test compound by use of a four-parameter logistic equation (Dotmatics, Bishops Stortford, United Kingdom).

### *In vitro* determination of persistence of action on bronchial epithelial cells.

The persistence of action of test agents was evaluated on immortalized bronchial epithelial cells (BEAS2B cells). Each experiment consisted of one nonwashout plate (96 wells) and a parallel washout plate, into which BEAS2B cells were seeded at a concentration of 3 × 10^4^ cells/well in growth medium (RPMI 1640, 2 mM l-glutamine, 10% fetal calf serum [FCS]) and then incubated for 24 h at 37°C and 5% CO_2_. Test and reference articles or undiluted DMSO (as vehicle) (0.5 μl/well) was added to the appropriate wells of the washout plate to give a final concentration of 0.5% DMSO. The plate was incubated for exactly 1 h at 37°C and 5% CO_2_. After the incubation time had elapsed, all wells on the washout plate were aspirated, and growth medium (100 μl/well) was added across the plate. After 24 h of incubation at 37°C, test and reference articles or undiluted DMSO (as vehicle) (0.5 μl/well) was added to the appropriate wells of the nonwashout plate to give a final concentration of 0.5% DMSO. The plate was incubated for exactly 1 h at 37°C and 5% CO_2_, after which A. fumigatus conidia were added across both plates to a final concentration of 1 × 10^3^/well. Fungal growth was determined after a further 24 h of incubation at 35°C and 5% CO_2_, by measuring galactomannan (GM) concentrations by use of Platelia GM-EIA kits (Bio-Rad Laboratories). The percent inhibition for each well was calculated, and the IC_50_ was calculated from the concentration-response curve generated for each test compound by use of a four-parameter logistic equation (Dotmatics, Bishops Stortford, United Kingdom).

### *In vivo* antifungal activity against A. fumigatus infection.

Specific-pathogen-free A/J mice (male, 5 weeks old) were purchased from Sankyo Labs Service Co. Ltd. (Tokyo, Japan) and adapted for 1 week in a temperature (24 ± 1°C)- and humidity (55% ± 5%)-controlled room under a 12-h day-night cycle. The mice were reared on a standard diet and tap water *ad libitum*. A/J mice were used for A. fumigatus infection and proved to be infected more efficiently, as described previously (44). Animals were then dosed with hydrocortisone (Sigma) (125 mg/kg of body weight administered subcutaneously) on days 3, 2, and 1 before infection and with cyclophosphamide (Sigma) (250 mg/kg administered intraperitoneally) 2 days before infection to induce temporary neutropenia, as previously reported (45). To avoid bacterial infection, drinking water was supplemented with tetracycline hydrochloride (Sigma) (1 μg/ml) and ciprofloxacin (Fluka) (64 μg/ml). A. fumigatus (ATCC 13073 [strain NIH 5233]; American Type Culture Collection, Manassas, VA) was grown on malt agar (Nissui Pharmaceutical, Tokyo, Japan) plates for 6 to 7 days at room temperature (24 ± 1°C). Conidia were aseptically dislodged from the agar plates and suspended in sterile distilled water with 0.05% Tween 80 and 0.1% agar. On the day of infection, conidial counts were assessed by use of a hemocytometer, and the inoculum was adjusted to obtain a concentration of 1.67 × 10^8^/ml in physiological saline. On day 0, 30 μl of the conidial suspension was administered intranasally.

Test agents suspended in physiological saline were administered daily intranasally (35 μl) on days 1 to 6, and the survival of animals was recorded for 7 days. The volume inserted intranasally has been reported to achieve almost 60% deposition into the lung (46). Deaths and the body weights of surviving animals were monitored daily. A body weight loss of >20% compared to an animal's weight on day 1 and mouse death were both defined as “dropout” events. Animals that lost >20% of their initial body weight were sacrificed. It was observed that A. fumigatus infection induced a “rolling” behavior, which was monitored and recorded (17). Bronchoalveolar lavage fluid (BALF) was collected on day 7 postinfection or on the day that the mouse dropped out of the study. The Aspergillus GM concentration in BALF was determined by use of Platelia GM-EIA kits (Bio-Rad Laboratories). The value was provided as the “cutoff index” (COI), which was calculated by the following formula: COI = OD in sample/OD in cutoff control (provided by the kit). All animal studies were approved by the Ethics Review Committee for Animal Experimentation of Nihon University. A. fumigatus studies were approved by the Microbial Safety Management Committee of the Nihon University School of Pharmacy (protocol E-H25-001).

### Statistical analysis.

Results are expressed as means ± standard errors of the means (SEM). Survival analysis was performed by Kaplan-Meier plotting followed by log rank (Mantel-Cox) tests, using the Prism 6 software program (GraphPad Software Inc., San Diego, CA). For comparison between groups, either ordinary one-way analysis of variance (ANOVA) with Tukey's *post hoc* comparison or Kruskal-Wallis ANOVA with Dunn's *post hoc* comparison was performed. Statistical significance was defined for *P* values of <0.05.

## Supplementary Material

Supplemental material
