# Supplementary material for: In Vitro and In Vivo Antifungal Profile of a Novel and Long-Acting Inhaled Azole, PC945, on Aspergillus fumigatus Infection
Source: Antimicrob Agents Chemother. 2017 Apr 24;61(5):e02280-16. doi: 10.1128/AAC.02280-16 (PMC5404542; doi:10.1128/AAC.02280-16)
Supplement: Supplemental material [file supp_61_5_e02280-16__index.html]

In Vitro and In Vivo Antifungal Profile of a Novel and Long-Acting Inhaled Azole, PC945, on Aspergillus fumigatus Infection — Supplemental material 

# *In Vitro* and *In Vivo* Antifungal Profile of a Novel and Long-Acting Inhaled Azole, PC945, on Aspergillus fumigatus Infection

## Supplemental material

- Supplemental file 1 -

  Supplemental Figures S1 and S2

  PDF, 409K
