# Supplementary material for: In Vitro and In Vivo Antifungal Profile of a Novel and Long-Acting Inhaled Azole, PC945, on Aspergillus fumigatus Infection
Source: Antimicrob Agents Chemother. 2017 Apr 24;61(5):e02280-16. doi: 10.1128/AAC.02280-16 (PMC5404542; doi:10.1128/AAC.02280-16)
Supplement: Supplemental material [file AAC.02280-16_zac005176125s1.pdf]

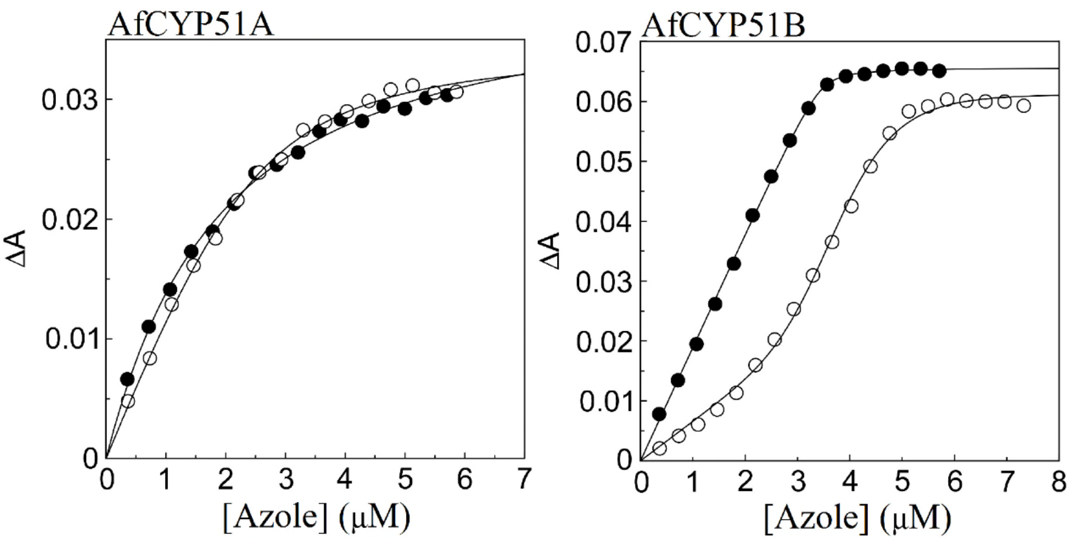

Supplementary Figure 1. Azole binding saturation curves for *A. fumigatus* CYP51A and CYP51B. Saturation curves were constructed from the absorbance difference $\Delta A_{peak\text{-}trough}$ of the type II difference spectra (Fig. 1B) for posaconazole (●) and PC945 (○). Each experiment was performed four times and PC945 binding to AfCYP51B six times, although only one replicate is shown.

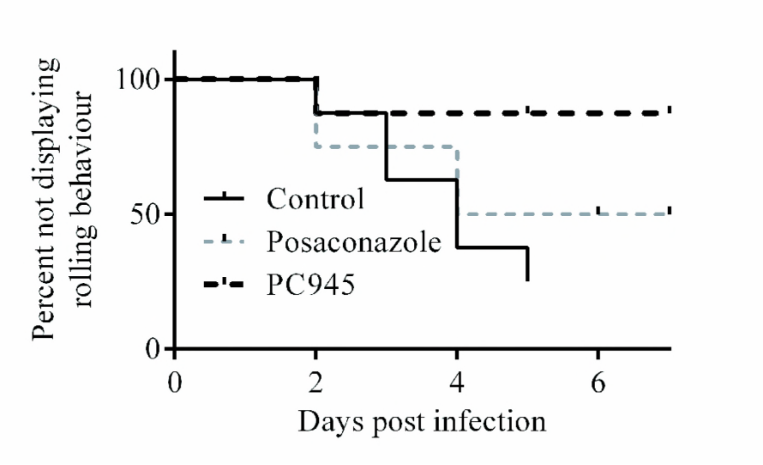

Supplementary Figure 2. Effect of once daily intranasal treatment of PC945 (14 µg/mouse) and posaconazole (14 µg/mouse) on *A. fumigatus* infection induced rolling behaviour in *A. fumigatus* infected immunocompromised mice

Y axis shows percentage of mice without displaying rolling behaviour. Some mice died or dropped out before displaying rolling behaviour.
